# Deletion of the non-adjacent genes *UL148* and *UL148D* impairs human cytomegalovirus-mediated TNF receptor 2 surface upregulation

**DOI:** 10.3389/fimmu.2023.1170300

**Published:** 2023-08-03

**Authors:** Vu Thuy Khanh Le-Trilling, Fabienne Maaßen, Benjamin Katschinski, Hartmut Hengel, Mirko Trilling

**Affiliations:** ^1^ Institute for Virology, University Hospital Essen, University of Duisburg−Essen, Essen, Germany; ^2^ Institute of Virology, Medical Center and Faculty of Medicine, University of Freiburg, Freiburg, Germany

**Keywords:** human cytomegalovirus (hcmv), tumor necrosis factor alpha (TNFα), TNF receptor 1 (TNFR1), TNF receptor 2 (TNFR2), ULb’, UL148, UL148D, TACE/ADAM17

## Abstract

Human cytomegalovirus (HCMV) is a prototypical β-herpesvirus which frequently causes morbidity and mortality in individuals with immature, suppressed, or senescent immunity. HCMV is sensed by various pattern recognition receptors, leading to the secretion of pro-inflammatory cytokines including tumor necrosis factor alpha (TNFα). TNFα binds to two distinct trimeric receptors: TNF receptor (TNFR) 1 and TNFR2, which differ in regard to their expression profiles, affinities for soluble and membrane-bound TNFα, and down-stream signaling pathways. While both TNF receptors engage NFκB signaling, only the nearly ubiquitously expressed TNFR1 exhibits a death domain that mediates TRADD/FADD-dependent caspase activation. Under steady-state conditions, TNFR2 expression is mainly restricted to immune cells where it predominantly submits pro-survival, proliferation-stimulating, and immune-regulatory signals. Based on the observation that HCMV-infected cells show enhanced binding of TNFα, we explored the interplay between HCMV and TNFR2. As expected, uninfected fibroblasts did not show detectable levels of TNFR2 on the surface. Intriguingly, however, HCMV infection increased TNFR2 surface levels of fibroblasts. Using HCMV variants and BACmid-derived clones either harboring or lacking the UL*b*’ region, an association between TNFR2 upregulation and the presence of the UL*b*’ genome region became evident. Applying a comprehensive set of UL*b*’ gene block and single gene deletion mutants, we observed that HCMV mutants in which the non-adjacent genes *UL148* or *UL148D* had been deleted show an impaired ability to upregulate TNFR2, coinciding with an inverse regulation of TACE/ADAM17.

## 1 Introduction

Human cytomegalovirus (HCMV, taxonomic name: Human herpesvirus 5; NCBI Taxonomy ID 10359) is the prototypical member of the *betaherpesvirinae*. A large proportion of the global human population is latently infected with HCMV as indicated by sero-prevalence rates ranging from 33.0 to 81.0% for developed countries and 59.1 to 95.7% for developing countries, which even further increase when the elderly are assessed (93.8 to 97.7% for developing countries) (1). With only very few exceptions (2), most primary and recurrent HCMV infections in healthy adults progress without overt disease. Conversely, HCMV frequently causes severe and often debilitating or even life-threatening diseases in individuals with immature, impaired, or senescent immunity such as congenitally infected newborns, transplant recipients, and AIDS patients (3). This direct association between clinical manifestations and impaired immunity, as well as several findings documenting how HCMV fundamentally shapes the immune system (4, 5), highlight the ongoing immunological battle between HCMV and its host. Accordingly, a comprehensive multi-parametric network analysis of numerous aspects of the immune system among twins of discordant HCMV sero-status showed that HCMV significantly affects 119 of 204 immunological parameters (6). Although the National Academy of Medicine already assigned the highest priority to the development of a HCMV vaccine in the year 2000 (7, 8), approved HCMV vaccines have remained unavailable. Besides the lack of defined surrogates of protective immunity, a major obstacle to vaccine development resides in the numerous potent modulators of immunity expressed by HCMV (5, 9) that target key aspects of the immune responses such as antigen presentation and T cell recognition (10), antibody responses (11), myeloid cells (12), NK cells (13), cytokine signaling, the interferon (IFN) system (14–17), and NFκB signaling initiated by tumor necrosis factor (TNF) receptor superfamily (TNFRSF) members (18). Accordingly, HCMV modulates various surface molecules and receptors on infected cells (see for example (19–21)).

In the case of TNF, the receptors, and down-stream signaling cascades, the mutual interplay between cytomegaloviruses and their hosts is particularly multifaceted going far beyond simple blockade. HCMV infection initially results in NFκB activation (see e.g. (22–24)), in part through the host-encoded casein kinase II (25). Accordingly, NFκB signaling is a key mediator in the transition of monocytes to an activated pro-inflammatory state upon HCMV infection (26). However, in cells in which HCMV productively replicates, IκBα degradation induced by exogenously added TNFα is significantly impaired, indicating the existence of viral inhibitors of NFκB signaling downstream of TNFR1 (27, 28). Accordingly, NFκB inhibitory activities have been shown for several HCMV-encoded gene products including pIE2-pp86 (24), cmvIL-10 (29), pUL44 (30), miR-US5-1 and miR-UL112-3p (31, 32), and pUL26 (33). Despite these HCMV-encoded antagonists of NFκB signaling, HCMV also benefits from certain NFκB activity (see. e.g. (34)). Accordingly, it is well known that the major immediate early promoter (MIEP) contains functional κB sites (35–37) and that TNFR1- and TNFR2-dependent signaling is capable to enhance the MIEP activity (38). Furthermore, viral genes expressed later during the replication cycle also benefit in terms of abundance from NFκB activity (34). In accordance with the notion that HCMV takes advantage from NFκB activity, the virus encodes proteins that activate NFκB activity such as pUL76 (39). HCMV even encodes transmembrane glycoproteins with clear sequence similarity to TNFRSF members such as the HVEM mimetic pUL144 (40), which binds BTLA (41, 42) and activates NFκB signaling through TRAF6 (43) and TAK supported by the ubiquitin ligase TRIM23 (44). We and others have shown that HCMV expresses the protein pUL138 that enhances surface levels of TNFR1 and thereby sensitizes pUL138-expressing cells to activate NFκB signaling at TNFα concentrations which are too low to activate cells devoid of pUL138 (27, 45, 46). Accordingly, in HCMV retinitis patients, increased levels of TNFR1 have been observed (47). Higher levels of soluble forms of TNFR1 have also been described during HCMV pneumonitis (48). Intriguingly, the latter work also described increased levels of TNFR2. Based on our observation that HCMV-infected cells exhibit an increased capacity to bind TNFα and aforementioned clinical data suggesting increased levels of soluble TNFR2, we interrogated here if and how HCMV affects TNFR2.

## 2 Materials and methods

### 2.1 Cells, viruses, and infection

Human MRC-5 fibroblasts (ATCC CCL-171, passages 10-18, male), the fibroblast cell line BJ-5ta (ATCC CRL-4001, male), RPE-1 (ATCC CRL-4000, female), HeLa (ATCC CCL-2, female), and HEK293T (ATCC CRL-11268, female) cells were grown in Dulbecco`s minimal essential medium (DMEM, Gibco) supplemented with 10% (v/v) fetal calf serum (FCS), penicillin, streptomycin, and 2 mM glutamine at 37°C in 5% CO_2_. U937 (ATCC CRL-1593.2, male) and THP-1 (ATCC TIB-202, male) cells were grown in Roswell Park Memorial Institute 1640 (RPMI-1640, Gibco) supplemented with 10% (v/v) FCS, penicillin, streptomycin, and 2 mM glutamine at 37°C in 5% CO_2_.

The following HCMV strains were used: AD169varS (corresponding to AD169varATCC), AD169varL (27), the bacterial artificial chromosome (BAC)-derived AD169 variants AD169varL and AD169varS reconstituted from the BAC clones AD169-BAC2 (27) and AD169-BAC20 (49), respectively, Towne (ATCC VR-977), and the endotheliotropic strain TB40/E, reconstituted from its respective BAC clone TB40-BAC4 (50). All BAC-derived viruses lack the US2-6 genome region that was replaced by the BAC cassette. Virus stocks were prepared as described previously by propagating HCMV in MRC-5 fibroblasts (51). HCMV infection was enhanced by centrifugation at 900 g for 30 min. HCMV stocks were titrated on MRC-5 fibroblasts.

The HSV-1 strain F was kindly provided by David Johnson (Portland, USA). Vaccinia virus (VACV) strain Western Reserve (WR) was originally provided by Bernard Moss (National Institutes of Health, Bethesda, MD).

### 2.2 Cytokines and inhibitors

The activation of NFκB signalling was induced by treatment with TNFα (R&D Systems). For the detection of IκBα degradation, cells were incubated with 20 ng/ml TNFα for 30 min. TNFα-induced gene expression was analyzed after 3 h of treatment. Ganciclovir (50 μM; Sigma) was used to prevent herpesviral DNA replication and decrease *late* gene expression. GCV was added at 2 h post-infection after removal of the virus-containing infection solution.

### 2.3 Generation of recombinant HCMV mutants

Recombinant HCMV mutants were generated according to previously published procedures (52, 53) using AD169-BAC2 or AD169-BAC20. For the construction of the HCMV deletion mutants, a PCR fragment was generated (see **Supplementary Table S1** for primer sequences) using the plasmid pSLFRTKn (54) as the template DNA. PCR fragments containing a kanamycin resistance gene were inserted into the parental BAC by homologous recombination in *E. coli*. The inserted cassette replaces the target sequence which was defined by flanking sequences in the primers. This cassette is flanked by frt-sites which can be used to remove the kanamycin resistance gene by flp-mediated recombination. The removal of the cassette results in a single remaining frt-site. For the generation of AD169varSΔgpt-UL148D-HA, an AD169-BAC20-based mutant expressing C-terminally hemagglutinin [HA]-tagged UL148D, the gpt sequence of the BAC cassette was replaced by a kanamycin cassette. Subsequently, the kanamycin cassette was removed, generating an frt-site that was used to introduce the UL148D-HA expression cassette, an frt-site-flanked fragment encompassing the cellular EF1 promoter in front of the UL148D-HA coding sequence. Correct mutagenesis of recombinant HCMV BACs was confirmed by southernblot and PCR analysis (data not shown). Recombinant HCMVs were reconstituted from HCMV BAC DNA by Superfect (Qiagen) or FugeneHD (Promega) transfection into permissive MRC-5 cells by following the instructions of the manufacturer. UL148D-HA expression by AD169varSΔgpt-UL148D-HA was confirmed by immunoblot analysis (data not shown).

### 2.4 Cloning of expression vectors and transient transfection

For the cloning of the UL148 and UL148D expression constructs, primers containing restriction sites and C-terminal HA epitope tag were used to amplify the respective gene product from AD169-BAC2 DNA. PCR fragments were cloned into pIRES2-EGFP by use of the introduced restriction sites. Plasmids were confirmed by the sequence determination of the inserted fragment. HeLa cells were transfected with pIRES2-EGFP plasmids expressing UL148-HA or UL148D-HA using FugeneHD (Promega) by following the manufacturer’s instructions.

### 2.5 Flow cytometry

For flow cytometry, cells were detached using Alfazyme (PAA Laboratories), washed, and incubated in 50 μl of 3% (v/v) FCS in phosphate-buffered saline (PBS) with labeled antibody (phycoerythrin [PE]-conjugated) anti-TNFR1, anti-TNFR2, or the respective PE-conjugated isotype control (R&D Systems) for 45 min on ice in the dark. For the quantification of TACE/ADAM17 surface densities, cells were incubated with an antibody detecting the TACE ectodomain (MAB9301, R&D Systems) followed by the incubation with a PE-conjugated secondary antibody (Biolegend). After antibody staining, cells were washed three times and measured in the PE channel of a FACS Canto II (Becton Dickinson). For detection of TNFα binding, cells were incubated with TNFα-Biotin and Streptavidin-FITC or let unstained. Cells were washed three times and measured in the FACS Canto II FITC channel. Histograms were generated by the use of FlowJo (Tree Star).

### 2.6 Immunoblot analysis

Immunoblotting was performed according to standard procedures. Briefly, cells were washed in PBS and lysed in lysis buffer (50 mM Tris-HCl [pH 7.5], 150 mM NaCl, 1% [vol/vol] IGEPAL [Sigma], 1% [vol/vol] Na-deoxycholate, 0.1% [vol/vol] SDS, 1 mM DTT, 0.2 mM PMSF, 1 μg/ml leupeptin and pepstatin, 50 mM NaF, 0.1 mM Na-vanadate, and Complete protease inhibitor [Roche]). Equal amounts of protein were subjected to SDS-PAGE and transferred to nitrocellulose membranes. Immunoblot analysis was performed using mouse monoclonal antibody (MAb) anti-β-actin (Sigma), mouse MAb recognizing HCMV pUL83/pp65 (3A12; Abcam), and rabbit polyclonal anti-IκBα (sc-371; Santa Cruz). The proteins were visualized using peroxidase-coupled secondary antibodies (Dianova) and the ECL chemiluminescence system (Cell Signaling).

### 2.7 Northern blotting and semi-quantitative RT-PCR analysis of specific transcripts

Total RNA was extracted from cells using the RNeasy minikit (Qiagen). Total RNA was subjected to morpholinepropanesulfonic acid (MOPS) gel electrophoresis and transferred to nylon membranes using a TurboBlotter (Schleicher and Schuell). Probes were prepared by PCR with gene-specific primers (see **Supplementary Table S2** for primer sequences) and digoxigenin-labeled dUTP (Roche) for the detection of the indicated transcripts. Hybridization and detection were performed as described by Roche manuals. For semi-quantitative RT-PCR, total RNA was digested with DNase I and used as the template for one-step RT-PCR (Qiagen) with gene-specific primers (see **Supplementary Table S2** for primer sequences).

### 2.8 Statistical analysis

Statistical significance was determined using an one-way ANOVA test as indicated in the figure legends. A p value of <0.05 was considered statistically significant. *, p value <0.05. **, p value <0.01. ***, p value <0.001. Calculations of p values were performed using GraphPad Prism.

## 3 Results

HCMV-AD169 was the first HCMV strain that was sequenced and annotated and that was, and still is, used for numerous studies. The AD169varATCC genome differs from clinical isolates by its lack of the UL*b*′ region. However, HCMV-AD169 variants were identified that retained large parts of the UL*b*′ region. AD169 variants with long (AD169varL) and short (AD169varS) UL genome regions differ concerning 11.8 kb of coding capacity within the UL*b*′ region. We used the two representative BAC-derived clones AD169-BAC2 and AD169-BAC20, which genetically correspond to AD169varL and AD169varS, respectively (49). For convenience, we named the AD169-BAC2- and AD169-BAC20-derived viruses AD169varL and AD169varS, respectively.

**Figure 1:**
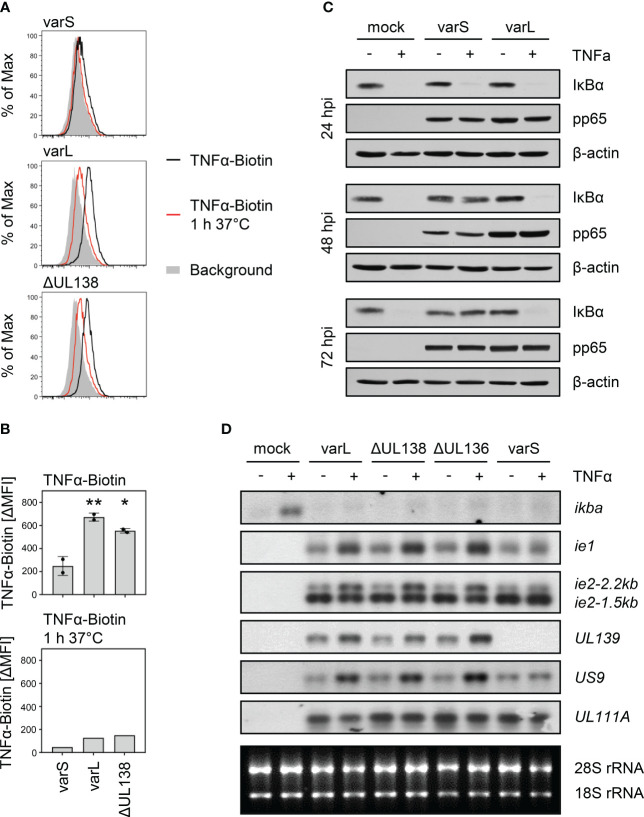
Positive association between the presence of the UL*b*’ gene region and TNFα binding as well as transcriptional upregulation of HCMV genes containing κB sites. **(A, B)** MRC-5 fibroblasts were infected (MOI 5) with HCMV AD169varS, AD169varL, or AD169varLΔUL138. At 72 h post-infection, cells were incubated with TNFα-Biotin for 1 h on ice and with Streptavidin-FITC for 30 min on ice and subsequently analyzed by flow cytometry. Histograms are shown in **(A)**. The difference between the mean fluorescence intensity (ΔMFI) of the TNFα-Biotin and the respective background signal was calculated and shown as a bars in **(B)**. The mean values +/- standard deviation (SD) as well as the values of individual experiments (n=2) are depicted. AD169varL and AD169varLΔUL138 infections were compared to AD169varS infection by one-way ANOVA. *, p<0.05. **, p<0.01. The second experiment also contained a control setting for which cells were treated with TNFα-Biotin for 1 h on ice and subsequently incubated for 1 h at 37°C before cells were incubated with Streptavidin-FITC for 30 min on ice (TNFα-Biotin, 1 h 37°C). **(C)** MRC-5 cells were mock treated or infected (MOI 5) with HCMV AD169varS or AD169varL. At 24, 48, and 72 h post-infection, cells were treated with 20 ng/ml TNFα for 30 min before protein lysates were generated for immunoblot analysis of indicated proteins. **(D)** MRC-5 cells were mock treated or infected (MOI 5) with HCMV AD169varL, AD169varLΔUL138, AD169varLΔUL136, or AD169varS. At 72 h post-infection, cells were treated with 20 ng/ml TNFα for 3 h and total RNA was prepared for northernblot analysis of indicated transcripts.

Based on previous findings regarding the upregulation of TNFR1 surface levels by the HCMV-encoded protein pUL138, we tested how HCMV affects the binding of biotinylated TNFα to infected fibroblasts. Upon ligand binding, TNFR1 and TNFR2 rapidly internalize (55, 56). Making use of this mechanism as a specificity control, cells were incubated for 1 h at 37°C which resulted in the expected decrease in the signal intensity, most likely through the internalization of TNFα-bound receptor complexes (**Figures 1A, B**). Cells infected with the UL*b*’-positive AD169varL (described in (27)) showed a TNFα binding which clearly exceeded the binding of cells infected with AD169varS which lacks the UL*b*’ region (**Figures 1A, B**). The increased TNFα binding was only partially dependent on pUL138 as indicated by cells infected with a *UL138* deletion mutant (ΔUL138). We concluded that at least one additional gene product of the UL*b’* region enhances the binding of TNFα to HCMV-infected fibroblasts.

To study the signaling of TNFα in HCMV-infected cells, we first assessed the TNFα-mediated proteasomal degradation of the NFκB inhibitory protein IκBα (also known as NFKBIA), which represents an essential step of the canonical NFκB signaling. On protein level, IκBα degradation is a hallmark of canonical NFκB signaling, while on mRNA level, a subsequent *ikba* induction as negative feedback regulation indicates NFκB-dependent gene expression. In accordance with the interpretation that the UL*b’* region encodes gene products that increase TNFα-induced NFκB activation, we observed TNFα-induced IκBα degradation at 48 and 72 hpi in cells infected with the UL*b’*-positive AD169varL, but not in cells infected with the UL*b’*-negative AD169varS (**Figure 1C**). While the IκBα protein needs to be degraded to enable canonical NFκB signaling, *ikba* transcription is strongly induced, which returns the system to its initial state. Therefore, we studied the upregulation of the well-known NFκB-responsive host gene *ikba*. In accordance with aforementioned publications documenting an inhibition of TNFα-induced signaling in HCMV-infected cells (28), we observed that HCMV impaired the TNFα-induced upregulation of *ikba* transcripts (**Figure 1D**). This effect occurred irrespective of *UL138* and the UL*b’* region. Given that HCMV also harbors functional κB sites in its genome, for example in the MIEP, we tested if TNFα treatment may increase the transcription of such viral genes. A clear upregulation of *ie1* and *ie2* mRNA amounts in TNFα-treated cells infected with an UL*b’*-positive HCMV, but not in cells infected with an HCMV variant lacking the UL*b’* region, indicated that UL*b’* gene product(s) indeed enhance(s) the TNFα-induced signaling upstream of viral promoters containing functional κB sites. For a more general assessment, we inspected the HCMV genome for additional κB sites in the vicinity of promoter regions. Among others (data not shown), we found such sites adjacent to the genes *US9* and *UL139*. Corroborating the notion that TNFα enhances the expression of HCMV-encoded genes harboring κB sites in an UL*b*’ gene region-dependent manner, we observed an upregulation of *US9* and *UL139* mRNA amounts by TNFα in cells infected with the UL*b’*-positive AD169varL but not in case of the UL*b’*-negative AD169varS (**Figure 1D**). Since deletions of *UL138* or *UL136* did not abrogate this effect, we concluded that the UL*b’* region codes for yet unidentified positive regulators of TNFα binding which enhance the activity of κB-containing HCMV promoters including the MIEP.

**Figure 2:**
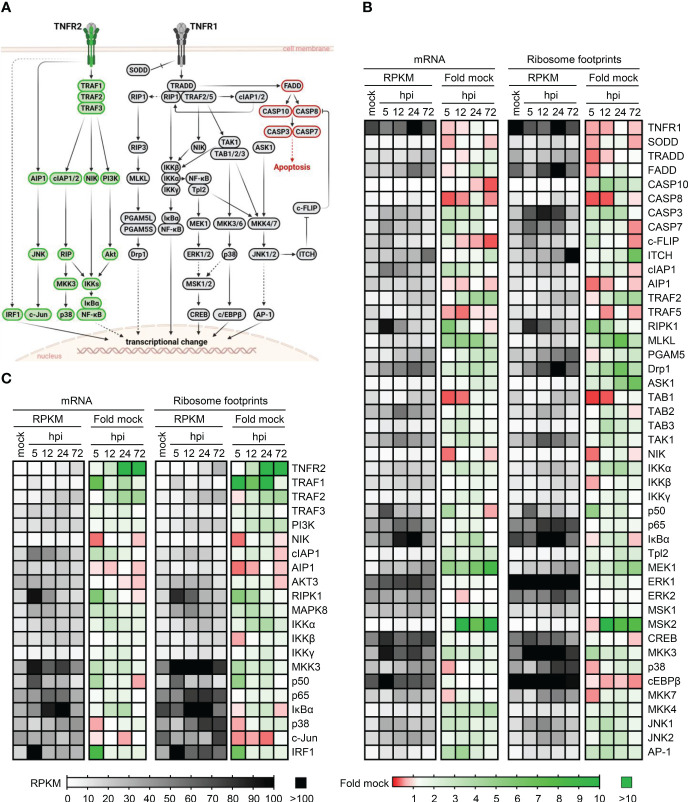
Transcription and ribosome occupancy of genes coding for components of TNFR1 and TNFR2 signaling during HCMV infection. **(A)** Simplified schema of the TNFR1 and TNFR2 signaling cascades according to the KEGG database (57). Proteins involved in TNFR2 signaling are depicted in green, TNFR1 signaling factors are shown in grey. Components involved in TNFR1-dependent apoptosis are marked in red. The schema was created with BioRender.com. **(B, C)** Publicly available data published in (58) were inspected regarding the transcription and ribosome occupancy at indicated time points during the course of HCMV infection. The heatmaps show the actual mRNA abundance (in RPKM) and the fold change compared to uninfected control cells. **(B)** shows the components of TNFR1 signaling and **(C)** shows the components of TNFR2 signaling.

The signaling pathways downstream of TNFR1 and TNFR2 are only partially overlapping (**Figure 2A**). One fundamental difference is the absence of a death domain in TNFR2 through which TNFR1 induces TRADD/FADD-dependent caspase activation. We inspected publicly available data sets (58) regarding HCMV-induced changes in transcript abundance and ribosome footprints of all components of the TNFR1 (**Figure 2B**) and TNFR2 (**Figure 2C**) downstream signaling listed in the *TNF signaling pathway - Homo sapiens (human)* of the KEGG database (“hsa04668” (57)). Since we were interested in both the transcript abundance of these factors and HCMV-induced alterations, we plotted RPKM as well as fold changes. Intriguingly, we observed that various mRNAs coding for mediators of TNFR2 signaling are continuously transcribed and appear to be translated (as indicated by the ribosome footprints) after HCMV infection, and that HCMV even enhances the abundance and ribosome occupancy of mRNAs coding for factors inherently involved in TNFR2 signaling such as TRAFs, and IKKs (**Figure 2C**). Conversely, TRADD, FADD, and Caspase 8, which are involved in TNFR1 signaling, show an opposite regulation (**Figure 2B**).

**Figure 3:**
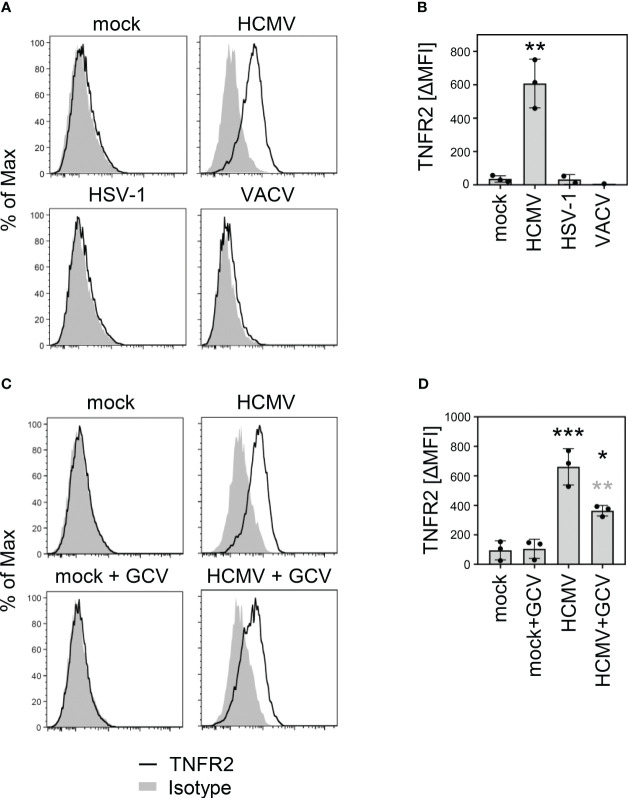
HCMV infection upregulates surface levels of TNF receptor 2. **(A)** MRC-5 cells were mock treated or infected (MOI 5) with HCMV AD169varL, HSV-1, or VACV. At 72 h post-infection (HCMV) or 24 h post-infection (HSV-1 and VACV), cells were stained with anti-TNFR2 or isotype control antibody and analyzed by flow cytometry. **(B)** The difference between the mean fluorescence intensity (ΔMFI) of the TNFR2 and the respective background signal was calculated and shown as bars. The mean values +/- SD as well as the values of individual experiments (n=1-3) are depicted. Infections were compared to the mock condition by one-way ANOVA. **, p<0.01. **(C)** MRC-5 cells were mock treated or infected with HCMV AD169varL (MOI 5). HCMV DNA replication and the accompanied late gene expression was prevented by the use of ganciclovir (GCV, 50 μM). At 72 h post-infection, cells were stained with anti-TNFR2 or isotype control antibody and analyzed by flow cytometry. **(D)** The difference between the mean fluorescence intensity (ΔMFI) of the TNFR2 and the respective background signal was calculated and shown as bars. The mean values +/- SD as well as the values of individual experiments (n=3) are depicted. Significance was calculated by one-way ANOVA. Black asterisks, compared to mock. Grey asterisks, compared to untreated condition. *, p<0.05. **, p<0.01. ***, p<0.001.

We observed an increased TNFα binding and an induction of HCMV genes harboring κB sites in cells infected with UL*b’*-positive HCMVs together with continuous or even enhanced expression of various mediators of TNF receptor signaling. Therefore, we considered it more likely that the increased TNFα binding results from the upregulation of cellular TNFRSF members rather than virus-encoded decoy receptors. Although TNFRSF ligands and receptors form a complex and often converging network, TNFα is only recognized by TNFR1 and TNFR2 (59). Since a relevant fraction of the upregulation of TNFα binding and most of the TNF-dependent upregulation of HCMV genes harboring κB sites was observed irrespective of the TNFR1 modulator pUL138, we analyzed surface levels of TNFR2. Contradicting a common belief that TNFR2 expression is restricted to immune cells, we observed a strong upregulation of TNFR2 on the surface of HCMV-infected MRC-5 cells (**Figure 3A**). This TNFR2 upregulation was not a general phenomenon elicited by DNA viruses because it was not observed when the cells were infected with herpes simplex virus (HSV)-1 or vaccinia virus (VACV) (**Figures 3A, B**). Treatment with ganciclovir (GCV), that inhibits the viral genome replication and drastically reduces the expression of viral *late* genes, which is functionally linked to genome amplification, resulted in intermediate TNFR2 surface levels (**Figures 3C, D**), indicating that at least one of the causative HCMV-encoded gene products is expressed in part with *early* (or even earlier kinetics) and in part with GCV-sensitive *late* expression profiles.

**Figure 4:**
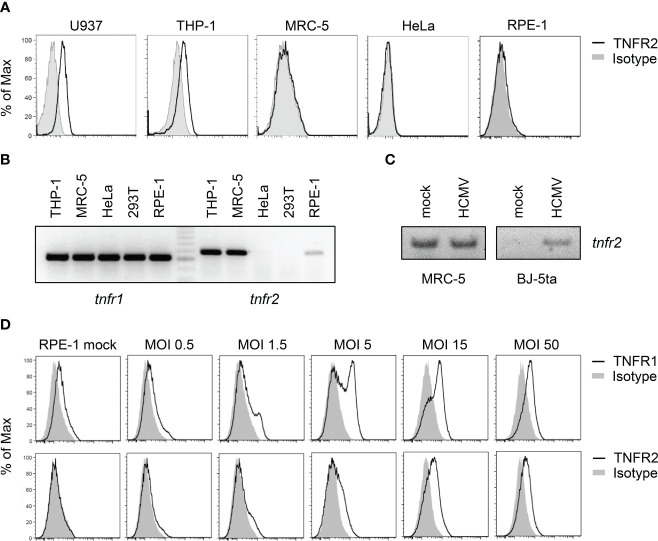
Cell lines differ in their expression of TNFR2. **(A)** Indicated cell lines were stained with anti-TNFR2 or isotype control antibody and analyzed by flow cytometry. **(B)** Total RNA was extracted from indicated cells and treated with DNase I. RT-PCR analysis was performed using *tnfr1*- and *tnfr2*-specific primers and 100 ng total RNA as the template. **(C)** MRC-5 and BJ-5ta fibroblasts were mock treated or infected with HCMV AD169varL (MOI 5). At 72 h post-infection, total RNA was prepared and treated with DNase I. RT-PCR analysis was performed using *tnfr2*-specific primers and 20 ng total RNA as the template. **(D)** RPE-1 epithelial cells were mock treated or infected with indicated virus doses of TB40-HCMV. Please note that calculations of MOIs are based on MRC-5 titers. At 96 h post-infection, cells were stained with anti-TNFR1, anti-TNFR2, or the respective isotype control antibody and analyzed by flow cytometry.

In the absence of HCMV infection, U937 and THP-1 cells, as expected, showed TNFR2 on the surface, whereas MRC-5, HeLa, and retinal pigment epithelial (RPE)-1 cells did not (**Figure 4A**). Intriguingly, however, uninfected MRC-5 and to a lesser extend RPE-1 cells expressed *tnfr2* mRNA (**Figure 4B**), suggesting that TNFR2 surface levels in MRC-5 cells are negatively regulated, at least in part, by post-transcriptional mechanisms that are altered upon HCMV infection. Accordingly, RT-PCR analysis using graded mRNA template dilutions showed that HCMV does not upregulate the *tnfr2* mRNA in MRC-5 cells (data not shown), indicating that the increase in TNFR2 surface levels of infected MRC-5 fibroblasts cannot be explained by an HCMV-induced transcriptional upregulation. Since *tnfr2* mRNA levels were increased upon HCMV infection in the data set published by Tirosh et al., who had infected human foreskin fibroblasts (HFF), while we did not observe an upregulation in MRC-5 cells, we tested another HCMV-permissive fibroblast cell line. In BJ-5ta, HCMV induced *tnfr2* transcription, indicating that our analysis was capable to recognize transcriptional differences, but that MRC-5 cells are special with regard to their baseline *tnfr2* transcription (**Figure 4C**, see a potential explanation in the discussion). Despite these transcriptional differences in uninfected cells, HCMV encounters *tnfr2* transcripts in all assessed fibroblasts, either constitutively expressed (MRC-5) or conditionally induced upon infection (BJ-5ta). Accordingly, TNFR2 surface levels are upregulated by HCMV in MRC-5 (e.g., **Figure 3**) and in BJ-5ta cells (see below).

To test whether the HCMV-induced TNFR2 upregulation occurs virus dose-dependently, we applied graded TB40-HCMV doses to RPE-1 cells, which express relatively low levels of *tnfr2* mRNA amounts (**Figure 4B**). Indeed, we observed a strong and multiplicity of infection (MOI)-dependent upregulation of TNFR1 and TNFR2 surface levels (**Figure 4D**).

**Figure 5:**
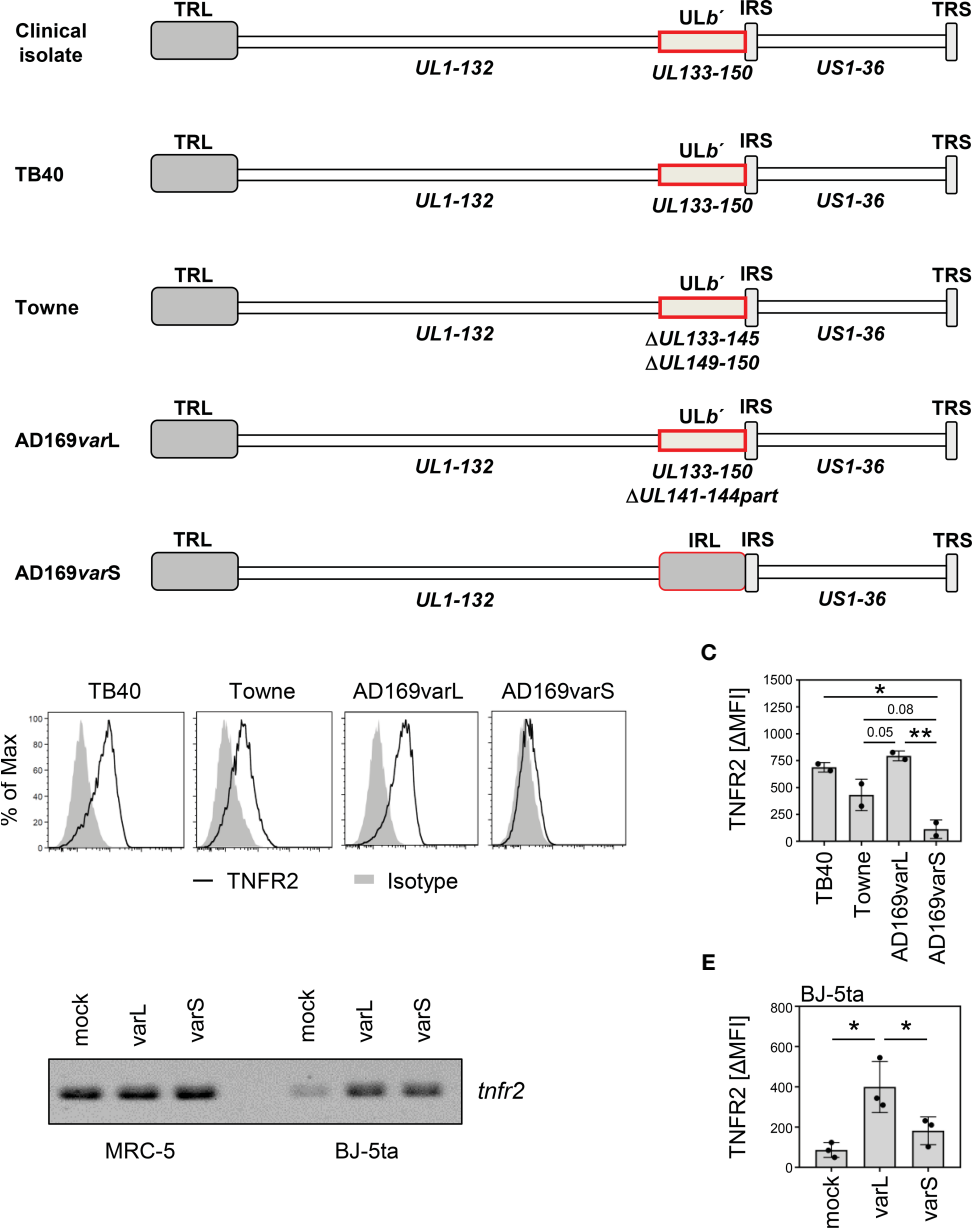
The HCMV UL*b′* region encodes modulators of TNFR2 surface levels. **(A)** Schematic overview of genome organization of indicated HCMV strains. UL, unique long; US, unique short; TRL, terminal repeat long; IRS, internal repeat short; TRS, terminal repeat short; IRL, internal repeat long; RL, repeat long. **(B)** MRC-5 fibroblasts were infected (MOI 5) with TB40-HCMV, Towne-HCMV, AD169varL-HCMV, or AD169varS-HCMV. At 72 h post-infection, cells were stained with anti-TNFR2 or isotype control antibody and analyzed by flow cytometry. **(C)** The difference between the mean fluorescence intensity (ΔMFI) of TNFR2 and the respective isotype control signal was calculated and shown as bars. The mean values +/- SD as well as the values of individual experiments (n=2) are depicted. Significance was calculated by one-way ANOVA. *, p<0.05. **, p<0.01. **(D)** MRC-5 and BJ-5ta cells were mock treated or infected with AD169varL or AD169varS (MOI 5). At 72 h post-infection, total RNA was prepared and treated with DNase **(I)** RT-PCR analysis was performed using *tnfr2*-specific primers and 10 ng total RNA as the template. **(E)** BJ-5ta cells were mock treated or infected with AD169varL or AD169varS (MOI 5). At 96 h post-infection, cells were stained with anti-TNFR2 or isotype control antibody and analyzed by flow cytometry. The difference between the mean fluorescence intensity (ΔMFI) of TNFR2 and the respective isotype control signal was calculated. The mean values +/- SD as well as the values of individual experiments (n=3) are depicted. Significance was calculated by one-way ANOVA. *, p<0.05.

To identify the genetic determinant(s) of the HCMV-mediated TNFR2 upregulation, we compared a set of HCMV strains that differ, among other aspects, in the degree to which the UL*b’* region is still present in the genome (**Figure 5A**). While TB40-HCMV, which harbors the entire UL*b*’ region, and AD169varL, which harbors most of the UL*b*’ region, upregulated TNFR2 surface densities, the UL*b’*-negative AD169varS failed to do so (**Figures 5B, C**). Interestingly, Towne-HCMV, which lacks parts of the UL*b*’ region, showed an intermediate TNFR2 upregulation phenotype (**Figures 5B, C**), suggesting that more than one UL*b*’ gene product influences the regulation of TNFR2. Since MRC-5 and BJ-5ta fibroblasts differ with regard to the baseline *tnfr2* transcription (**Figures 4C**, **5D**), we also assessed the level of *tnfr2* transcripts and TNFR2 protein surface levels in BJ-5ta cells infected either with AD169varL or AD169varS (**Figures 5D, E**). Irrespective if cells express *tnfr2* constitutively (MRC-5) or conditionally upon HCMV infection (BJ-5ta), UL*b*’-positive HCMVs (AD169varL) strongly enhanced TNFR2 surface levels, while UL*b*’-negative HCMVs (AD169varS) did not.

**Figure 6:**
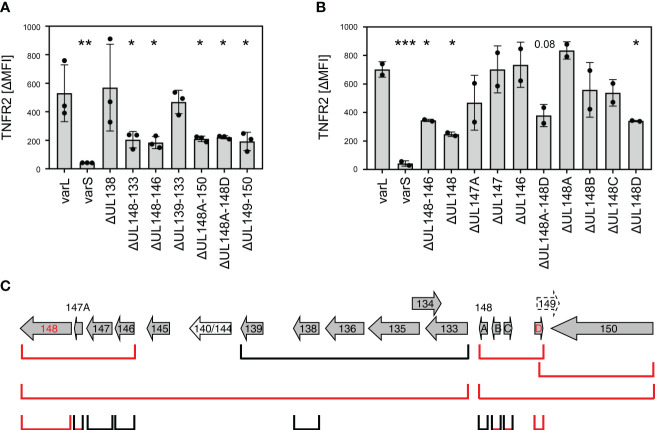
Deletion of *UL148* or *UL148D* impairs HCMV-mediated TNFR2 upregulation. **(A, B)** MRC-5 fibroblasts were infected (MOI 5) with indicated HCMV mutants. At 72 h post-infection, cells were stained with anti-TNFR2 or isotype control antibody and analyzed by flow cytometry. The difference between the mean fluorescence intensity (ΔMFI) of TNFR2 and the respective isotype control signal was calculated. Pooled data of three **(A)** or two **(B)** infection experiments are shown. The mean values +/- SD as well as the values of individual experiments are depicted. All mutant infections were compared to the AD169varL infection by one-way ANOVA. *, p<0.05. **, p<0.01. ***, p<0.001. **(C)** Schema of the canonical AD169 UL*b′* ORFs. Please note that the order of the UL*b′* genes in the AD169 genome does not match with the UL nomenclature. The ORFs that are deleted in the respective HCMV mutants are schematically depicted. Mutants with decreased TNFR2 signals are marked in red.

To identify genes that are essential for the TNFR2 upregulation, we generated and tested a comprehensive panel of gene block and single gene deletion HCMV mutants. Starting with bigger deletions, we confirmed that indeed at least two gene products influence the upregulation of TNFR2, as indicated by the intermediate phenotypes of the two non-overlapping deletions ΔUL148-133 and UL148A-UL150 (**Figure 6A**). By iterative cycles of gene (block) deletion in HCMV by BACmid mutagenesis and functional TNFR2 analysis, we further narrowed down the responsible gene loci. While the gene region spanning from *UL139* to *UL133* was dispensable for TNFR2 upregulation, the regions *UL148* to *UL146* and *UL148A* to *UL148D* were not (**Figure 6A**). Using a set of single gene deletions of the affected canonical genes, we found that deletion of *UL148* impaired the HCMV-mediated upregulation of TNFR2 (**Figures 6B, C**). We assume that the slight effect observed with the *UL147A* deletion may result from a deregulation of the adjacent *UL148* gene (**Figures 6B, C**). The observation that the ΔUL148A-UL148D mutant as well as the ΔUL149-UL150 mutant showed an effect (**Figure 6A**) suggested that the gene *UL148D*, which was affected in both gene block mutants, may be involved in TNFR2 upregulation. This assumption turned out to be true as indicated by the loss of function observed with a specific *UL148D* HCMV deletion mutant (**Figures 6B, C**). Again, we assume that partial effects observed by deletions of *UL148B* and *UL148C* may due to do indirect effects on adjacent genes. Taken together, *UL148*, located close to the UL region, and *UL148D*, located close to the RS region, are required for the HCMV-mediated upregulation of TNFR2 in fibroblasts. Since Towne-HCMV harbors the *UL148* gene but lacks *UL148D*, this argumentation is in agreement with the intermediate TNFR2 upregulation phenotype observed for Towne-HCMV (**Figures 5B, C**). Our data document that HCMV encodes at least two non-adjacent genes in the UL*b*’ region that are indispensable for a post-transcriptional upregulation of TNFR2 surface levels.

**Figure 7:**
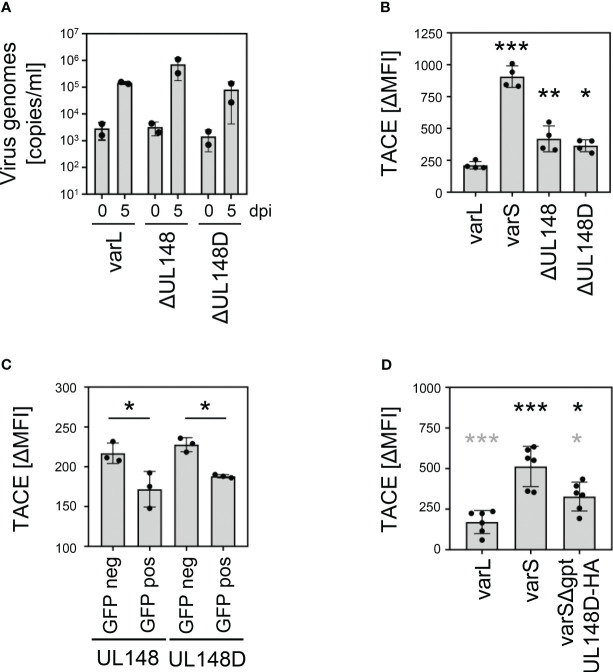
Regulation of TACE by pUL148 and pUL148D. **(A)** MRC-5 cells were infected with HCMV AD169varL, AD169varLΔUL148, or AD169varLΔUL148D (MOI 0.05). At 2 h post-infection, fresh medium was added after removal of the virus-containing infection solution. At 0 and 5 days post-infection, supernatant of infected cells was collected and DNA was prepared for quantification of supernatant HCMV genomes by diagnostic qRT-PCR. The mean values +/- SD as well as the values of individual experiments (n=2) are depicted. **(B)** MRC-5 cells were infected (MOI 5) with HCMV AD169varL, AD169varLΔUL148, or AD169varLΔUL148D. At 72 h post-infection, cells were stained with anti-TACE and analyzed by flow cytometry. The difference between the mean fluorescence intensity (ΔMFI) of TACE and the respective background signal was calculated. The mean values +/- SD as well as the values of individual infections (n=4) are shown. The mutant infections were compared to the AD169varL infection by one-way ANOVA. *, p<0.05. **, p<0.01. ***, p<0.001. **(C)** HeLa cells were transfected with UL148 and UL148D pIRES2-EGFP expression plasmids. At 24 h post-transfection, cells were harvested for flow cytometry. GFP-negative and GFP-positive cells were analyzed for TACE surface density. The difference between the mean fluorescence intensity (ΔMFI) of TACE and the respective background signal was calculated. The mean values +/- SD as well as the values of individual transfections (n=4) are shown. Significance was calculated by one-way ANOVA. *, p<0.05. **(D)** MRC-5 cells were infected (MOI 5) with HCMV AD169varL, AD169varS, or AD169varSΔgpt-UL148D-HA. At 72 h post-infection, cells were stained with anti-TACE and analyzed by flow cytometry. The difference between the mean fluorescence intensity (ΔMFI) of TACE and the respective background signal was calculated. The mean values +/- SD as well as the values of individual infections (n=6) are shown. Significance was calculated by one-way ANOVA. Black asterisks, compared to AD169varL infection. Grey asterisks, compared to AD169varS infection. *, p<0.05. ***, p<0.001.

Studies by Wang et al. and confirmed by Nguyen et al. showed that pUL148 interacts with CD58/LFA-3 and retains it intracellularly (60, 61). The work by Wang et al. made comprehensive mass-spectrometry data publicly available (61), which may reveal other pUL148 targets. Only a non-significant trend towards TNFR2 downregulation in the absence of *UL148* was observed (0.82-fold; p~0.22), maybe due to the presence of pUL148D. Intriguingly, the supplementary data shows an increase of *disintegrin and metalloproteinase domain-containing protein 17* (ADAM17; ΔUL148/wt fold ratio: 4.05; supplementary data of (61)), which is also known as *tumor necrosis factor-α-converting enzyme* (TACE). TACE induces TNFR2 shedding (62, 63). Based on our TNFR2 findings and aforementioned published work, we tested if the upregulation of TNFR2 levels by pUL148 and pUL148D may be explained through decreased levels of TACE. Compared to the parental AD169varL, HCMV mutants lacking the capacity to express pUL148 or pUL148D replicated to similar levels in fibroblasts (**Figure 7A**). With regard to TACE, however, AD169varL significantly decreased surface levels compared to AD169varS, and ΔUL148 and ΔUL148D showed intermediate phenotypes (**Figure 7B**). Conversely, ectopic expression of UL148 or UL148D in HeLa cells significantly decreased TACE levels (**Figure 7C**), and the insertion of the UL148D gene into the UL*b*’-negative AD169varS restored the intermediate phenotype of TACE downregulation (varSΔgpt-UL148D-HA, **Figure 7D**), suggesting that the herein described TNFR2 upregulation mediated by pUL148 and pUL148D is caused, at least in part, by their capacity to downregulate TACE.

## 4 Discussion

Here, we show that HCMV actively stimulates the surface disposition of TNFR2 on infected fibroblasts. This seeding observation was not restricted to our laboratory. After we knew what to look for, we recognized that supplementary data sets accompanying publications of our colleagues support our observation regarding the HCMV-induced TNFR2 upregulation. Although not mentioned at all in the article, the associated supplementary data by Weekes et al. also indicates a significant upregulation of TNFR2 at the plasma membrane (see **Supplementary Table 2** of (21)). The fact that the UL*b*’-positive HCMV strain Merlin was used and primary human foreskin fibroblasts were infected while we used AD169varL and TB40-HCMV infections of MRC-5 and RPE-1 cells, respectively, confirms and generalizes the HCMV-induced TNFR2 upregulation. Intriguingly, we found that this upregulation is strictly associated with the presence of the UL*b’* gene region, which becomes increasingly established as central hub for the regulation of the members of the TNF receptor superfamily and their ligands.

A surprising side observation was the abundant expression of *tnfr2* mRNAs in MRC-5 cells, which did not result in detectable TNFR2 surface levels in the absence of HCMV infection. The promoter of the human *tnfr2* gene comprises various enhancer elements that respond to numerous transcription factors such as STATs, IRFs, and AP-1, which may explain the expression (see e.g. (64, 65)). In addition to immune cells such as regulatory T cells, mesenchymal stem cells (MSCs) express TNFR2 and require TNFR2 for their immune suppressive capacities (66, 67). Intriguingly, Zhang et al. recently noticed that MRC-5 cells share several properties with human umbilical cord-derived MSCs (hUC-MSCs) including the promotion of Tregs and the induction of the immunomodulatory molecule IDO in response to IFNγ and TNFα (68).

Using a comprehensive panel of HCMV UL*b*’ mutants, we found that deletion of *UL148* and *UL148D* impaired the HCMV-mediated upregulation of TNFR2. Intermediate TNFR2 levels were observed upon deletion of either *UL148* or *UL148D*, whereas the combined loss of both genes resulted in an additive loss of TNFR2 surface levels. The fact that HCMV devotes at least two genes to upregulate TNFR2 indicates an important biologic relevance. Both pUL148 and pUL148D are expressed with *early/late* kinetics (21) which is in line with our observation that GCV treatment decreased HCMV-induced TNFR2 upregulation. So far, functional characterization of the *UL148D* gene region mainly focused on the role of hcmv-miR-UL148D, which targets the *ERN1*, *ACVR1B*, *IEX1*, and *CCL5* mRNAs blocking apoptosis (69–73). In their global assessment of *RNA-induced silencing complex* (RISC)-associated mRNAs in Normal human dermal fibroblasts (NHDF) infected either with the UL*b*’-negative AD169varATCC or the UL*b*’-positive HCMV TR, Pavelin et al. did not observe a relevant change of the TNFRSF1B mRNA in RISC complexes (see supplementary data of (74)), arguing against HCMV-encoded or induced miRNAs directly targeting *tnfr2* mRNA. Although pUL148D is expressed (21) and is not disrupted in so far sequenced HCMV genomes (75), it awaits further in-depth molecular characterizations. In clear contrast to pUL148D, different groups investigated functions of pUL148. The Kamil laboratory showed that pUL148 is an ER-resident transmembrane glycoprotein which alters the tropism of HCMV (76). The pUL148 interacts with SEL1L, activates the unfolded protein response (77), reorganizes the ER (78), and influences ER-associated degradation (ERAD) (79). TNFR2-mediated signaling induces TRAF2 degradation by a mechanism relying on the ubiquitin E2 ligase Ubc6 (80), which is also important for the ubiquitination of target proteins in ERAD (81), and ER stress leads to increased TNFR2 levels based on an association with progranulin (82). This raises the intriguing question if the role of pUL148 in the ER and the TNFR2 upregulation are mechanistically coupled.

Wang et al. published mass-spectrometry data, for which cells had been infected with HCMV either expressing or lacking *UL148* (61). Among the top five hits of differentially regulated proteins, their supplementary data showed *disintegrin and metalloproteinase domain-containing protein 17* (ADAM17; ΔUL148/wt fold ratio: 4.05 (61)), which is also known as *tumor necrosis factor-α-converting enzyme* (TACE). TACE induces TNFR2 shedding (62, 63). For pUL148, we confirmed this down-regulation of TACE. Furthermore, we extended the mechanism of TACE down-regulation to pUL148D. Thus, the pUL148/pUL148D-mediated increase in TNFR2 surface levels may be explained, at least in part, through their decreasing effect on TACE.

One intriguing question is why HCMV regulates TNFR2. Apparently, it is not a general phenomenon of *betaherpesvirinae* because HHV-7 seems not to upregulate TNFR2 levels (83). The facts that HCMV does not replicate in mice, and that MCMV decreases surface levels of TNFR1 and TNFR2 (84) rule out *in vivo* experiments in mice to address immune suppressive aspects of the CMV-induced TNFR2 upregulation. TNFR1 and TNFR2 both recognize TNFα (see e.g (85)). For certain viruses, TNFα mediates its antiviral activity through TNFR1 rather than through TNFR2 (86, 87). Thus, TNFR2 upregulation as a sequestration mechanism diverting TNFα from TNFR1 to TNFR2 may appear as a plausible argument at first glance. However, we and others showed that HCMV encodes pUL138 which upregulates TNFR1 surface levels (27, 45, 46). Therefore, it is highly likely that additional reasons beyond withholding TNFα from TNFR1 drove HCMV to express at least two gene products upregulating TNFR2. In the case of HIV, TNFα signaling via TNFR2 inhibits the viral entry into primary tissue culture-differentiated macrophages (88, 89). For non-mammalian viruses, a role of fish TNFα favoring virus replication has been described (90). In case of the rhabdovirus SVCV, TNFα signals through its receptor TNFR2 to enhance viral replication (91). Thus, TNFR2 can in principle elicit antiviral and proviral signals. However, global CRISPRi and CRISPRn screens for host factors affecting HCMV infections (conducted with HCMV-Merlin in HFFs) neither showed significant advantages nor disadvantages regarding HCMV infection associated with a loss of TNFRSF1B coding capacity (see supplementary data set of (92)). This argues against direct effects of TNFR2 on HCMV replication in fibroblasts. Accordingly, a siRNA screen of factors modulating HCMV replication comprised TNFRSF1B but also did not observe a relevant increase or decrease in virus replication (see **Supplementary Tables 1–3** in (93)). The interpretation that HCMV does not require TNFR2 for replication in cell culture systems such as fibroblasts may explain why fibroblast-adapted HCMV strains such as AD169*var*S have lost the UL*b*’ region comprising the two TNFR2 regulators. If the TNFR2 upregulation would be critical for HCMV replication in fibroblasts, the replacement of the UL*b*’ region should have been counter selected.

In the absence of pathogens and tumors, TNFR2 expression is usually restricted to cells of the immune system. Accordingly, TNFR2-selective agonists have been mostly applied to lymphocytes (94, 95). It is not trivial to anticipate which TNFR2 downstream signaling events and gene expression changes occur in HCMV-permissive cells and might be advantageous for HCMV. However, TNFR2 “makes TNF a friend of tumors” (96), e.g., by protecting malignant cells from DNA damage (97). Furthermore, TNFR2 engagement on endothelial progenitor cells leads to increased expression of anti-inflammatory mediators such as IL-10, TGFβ, and HLA-G (98). Thus, the upregulation of TNFR2 on HCMV-infected cells appears as another example of immuno-regulatory functions that are shared by tumors and viruses. It will be interesting to investigate the exact proviral function(s) resulting from TNFR2 upregulation in the future – maybe by using herein described HCMV mutants.

## Data availability statement

The original contributions presented in the study are included in the article/**Supplementary Material**. Further inquiries can be directed to the corresponding author.

## Author contributions

VTKL-T, FM, and BK performed research. VTKL-T, FM, HH, and MT analyzed data. VTKL-T, HH, and MT interpreted data. VTKL-T and MT supervised the project. VTKL-T and MT wrote the manuscript. All authors contributed to the article and approved the submitted version.

## References

[1] Fowler K Mucha J Neumann M Lewandowski W Kaczanowska M Grys M. A systematic literature review of the global seroprevalence of cytomegalovirus: possible implications for treatment, screening, and vaccine development. BMC Public Health (2022) 22:1659. doi: 10.1186/s12889-022-13971-7 36050659 PMC9435408

[2] Rafailidis PI Mourtzoukou EG Varbobitis IC Falagas ME. Severe cytomegalovirus infection in apparently immunocompetent patients: a systematic review. Virol J (2008) 5:47. doi: 10.1186/1743-422X-5-47 18371229 PMC2289809

[3] Griffiths P Reeves M. Pathogenesis of human cytomegalovirus in the immunocompromised host. Nat Rev Microbiol (2021) 19:759–73. doi: 10.1038/s41579-021-00582-z PMC8223196 34168328

[4] Goodier MR Jonjic S Riley EM Juranic Lisnic V. CMV and natural killer cells: shaping the response to vaccination. Eur J Immunol (2018) 48:50–65. doi: 10.1002/eji.201646762 28960320

[5] Picarda G Benedict CA. Cytomegalovirus: shape-shifting the immune system. J Immunol (2018) 200:3881–9. doi: 10.4049/jimmunol.1800171 PMC5992493 29866770

[6] Brodin P Jojic V Gao T Bhattacharya S Angel CJ Furman D. Variation in the human immune system is largely driven by non-heritable influences. Cell (2015) 160:37–47. doi: 10.1016/j.cell.2014.12.020 25594173 PMC4302727

[7] Stratton KR Durch JS R.S. L. Vaccines for the 21st Century: A Tool for Decisionmaking. Washington (DC): National Academies Press (US) (2000). 25121214

[8] Schleiss MR Permar SR Plotkin SA. Progress toward Development of a Vaccine against Congenital Cytomegalovirus Infection. Clin Vaccine Immunol (2017) 24(12):e00268-17. doi: 10.1128/CVI.00268-17 29046308 PMC5717185

[9] Powers C DeFilippis V Malouli D Fruh K. Cytomegalovirus immune evasion. Curr Top Microbiol Immunol (2008) 325:333–59. doi: 10.1007/978-3-540-77349-8_19 18637515

[10] Halenius A Gerke C Hengel H. Classical and non-classical MHC I molecule manipulation by human cytomegalovirus: so many targets-but how many arrows in the quiver? Cell Mol Immunol (2015) 12:139–53. doi: 10.1038/cmi.2014.105 PMC4654289 25418469

[11] Corrales-Aguilar E Hoffmann K Hengel H. CMV-encoded Fcgamma receptors: modulators at the interface of innate and adaptive immunity. Semin Immunopathol (2014) 36:627–40. doi: 10.1007/s00281-014-0448-2 25288477

[12] Brinkmann MM Dag F Hengel H Messerle M Kalinke U Cicin-Sain L. Cytomegalovirus immune evasion of myeloid lineage cells. Med Microbiol Immunol (2015) 204:367–82. doi: 10.1007/s00430-015-0403-4 25776081

[13] Lisnic B Lisnic VJ Jonjic S. NK cell interplay with cytomegaloviruses. Curr Opin Virol (2015) 15:9–18. doi: 10.1016/j.coviro.2015.07.001 26208082

[14] Hengel H Koszinowski UH Conzelmann KK. Viruses know it all: new insights into IFN networks. Trends Immunol (2005) 26:396–401. doi: 10.1016/j.it.2005.05.004 15922665

[15] Marshall EE Geballe AP. Multifaceted evasion of the interferon response by cytomegalovirus. J Interferon Cytokine Res (2009) 29:609–19. doi: 10.1089/jir.2009.0064 PMC2743745 19708810

[16] Becker T Le-Trilling VTK Trilling M. Cellular cullin RING ubiquitin ligases: druggable host dependency factors of cytomegaloviruses. Int J Mol Sci (2019) 20(7):1636. doi: 10.3390/ijms20071636 30986950 PMC6479302

[17] Trilling M Le VT Hengel H. Interplay between CMVs and interferon signaling: implications for pathogenesis and therapeutic intervention. Future Microbiol (2012) 7:1269–82. doi: 10.2217/fmb.12.109 23075446

[18] Benedict CA Ware CF. Virus targeting of the tumor necrosis factor superfamily. Virology (2001) 289:1–5. doi: 10.1006/viro.2001.1109 11601911

[19] Gudleski-O'Regan N Greco TM Cristea IM Shenk T. Increased expression of LDL receptor-related protein 1 during human cytomegalovirus infection reduces virion cholesterol and infectivity. Cell Host Microbe (2012) 12:86–96. doi: 10.1016/j.chom.2012.05.012 22817990 PMC3405916

[20] Hsu JL Boomen DJvd Tomasec P Weekes MP Antrobus R Stanton RJ. Plasma membrane profiling defines an expanded class of cell surface proteins selectively targeted for degradation by HCMV US2 in cooperation with UL141. PloS Pathog (2015) 11:e1004811. doi: 10.1371/journal.ppat.1004811 25875600 PMC4397069

[21] Weekes MP Tomasec P Huttlin EL Fielding CA Nusinow D Stanton RJ. Quantitative temporal viromics: an approach to investigate host-pathogen interaction. Cell (2014) 157:1460–72. doi: 10.1016/j.cell.2014.04.028 PMC4048463 24906157

[22] Yurochko AD Huang ES. Human cytomegalovirus binding to human monocytes induces immunoregulatory gene expression. J Immunol (1999) 162:4806–16. doi: 10.4049/jimmunol.162.8.4806 10202024

[23] Kowalik TF Wing B Haskill JS Azizkhan JC Baldwin AS Jr. Huang ES. Multiple mechanisms are implicated in the regulation of NF-kappa B activity during human cytomegalovirus infection. Proc Natl Acad Sci USA (1993) 90:1107–11. doi: 10.1073/pnas.90.3.1107 PMC45820 8381532

[24] Taylor RT Bresnahan WA. Human cytomegalovirus IE86 attenuates virus- and tumor necrosis factor alpha-induced NFkappaB-dependent gene expression. J Virol (2006) 80:10763–71. doi: 10.1128/JVI.01195-06 PMC1641772 17041226

[25] Nogalski MT Podduturi JP DeMeritt IB Milford LE Yurochko AD. The human cytomegalovirus virion possesses an activated casein kinase II that allows for the rapid phosphorylation of the inhibitor of NF-kappaB, IkappaBalpha. J Virol (2007) 81:5305–14. doi: 10.1128/JVI.02382-06 PMC1900216 17344282

[26] Chan G Bivins-Smith ER Smith MS Yurochko AD. Transcriptome analysis of NF-kappaB- and phosphatidylinositol 3-kinase-regulated genes in human cytomegalovirus-infected monocytes. J Virol (2008) 82:1040–6. doi: 10.1128/JVI.00864-07 PMC2224586 18003728

[27] Le VT Trilling M Hengel H. The cytomegaloviral protein pUL138 acts as potentiator of tumor necrosis factor (TNF) receptor 1 surface density to enhance ULb'-encoded modulation of TNF-alpha signaling. J Virol (2011) 85:13260–70. doi: 10.1128/JVI.06005-11 PMC3233134 21976655

[28] Jarvis MA Borton JA Keech AM Wong J Britt WJ Magun BE. Human cytomegalovirus attenuates interleukin-1beta and tumor necrosis factor alpha proinflammatory signaling by inhibition of NF-kappaB activation. J Virol (2006) 80:5588–98. doi: 10.1128/JVI.00060-06 PMC1472148 16699040

[29] Nachtwey J Spencer JV. HCMV IL-10 suppresses cytokine expression in monocytes through inhibition of nuclear factor-kappaB. Viral Immunol (2008) 21:477–82. doi: 10.1089/vim.2008.0048 PMC2831744 19115937

[30] Fu YZ Su S Zou HM Guo Y Wang SY Li S. Human cytomegalovirus DNA polymerase subunit UL44 antagonizes antiviral immune responses by suppressing IRF3- and NF-kappaB-mediated transcription. J Virol (2019) 93(11):e00181-19. doi: 10.1128/JVI.00181-19 30867312 PMC6532097

[31] Landais I Pelton C Streblow D DeFilippis V McWeeney S Nelson JA. Human cytomegalovirus miR-UL112-3p targets TLR2 and modulates the TLR2/IRAK1/NFkappaB signaling pathway. PloS Pathog (2015) 11:e1004881. doi: 10.1371/journal.ppat.1004881 25955717 PMC4425655

[32] Hancock MH Hook LM Mitchell J Nelson JA. Human Cytomegalovirus MicroRNAs miR-US5-1 and miR-UL112-3p Block Proinflammatory Cytokine Production in Response to NF-kappaB-Activating Factors through Direct Downregulation of IKKalpha and IKKbeta. mBio (2017) 8(2):e00109-17. doi: 10.1128/mBio.00109-17 28270578 PMC5340867

[33] Mathers C Schafer X Martinez-Sobrido L Munger J. The human cytomegalovirus UL26 protein antagonizes NF-kappaB activation. J Virol (2014) 88:14289–300. doi: 10.1128/JVI.02552-14 PMC4249132 25275128

[34] DeMeritt IB Podduturi JP Tilley AM Nogalski MT Yurochko AD. Prolonged activation of NF-kappaB by human cytomegalovirus promotes efficient viral replication and late gene expression. Virology (2006) 346:15–31. doi: 10.1016/j.virol.2005.09.065 16303162 PMC2600890

[35] DeMeritt IB Milford LE Yurochko AD. Activation of the NF-kappaB pathway in human cytomegalovirus-infected cells is necessary for efficient transactivation of the major immediate-early promoter. J Virol (2004) 78:4498–507. doi: 10.1128/JVI.78.9.4498-4507.2004 PMC387686 15078930

[36] Prosch S Staak K Stein J Liebenthal C Stamminger T Volk HD. Stimulation of the human cytomegalovirus IE enhancer/promoter in HL-60 cells by TNFalpha is mediated via induction of NF-kappaB. Virology (1995) 208:197–206. doi: 10.1006/viro.1995.1143 11831701

[37] Lee Y Sohn WJ Kim DS Kwon HJ. NF-kappaB- and c-Jun-dependent regulation of human cytomegalovirus immediate-early gene enhancer/promoter in response to lipopolysaccharide and bacterial CpG-oligodeoxynucleotides in macrophage cell line RAW 264.7. Eur J Biochem (2004) 271:1094–105. doi: 10.1111/j.1432-1033.2004.04011.x 15009188

[38] Medvedev AE Sundan A Espevik T. Involvement of the tumor necrosis factor receptor p75 in mediating cytotoxicity and gene regulating activities. Eur J Immunol (1994) 24:2842–9. doi: 10.1002/eji.1830241139 7957575

[39] Costa H Nascimento R Sinclair J Parkhouse RM. Human cytomegalovirus gene UL76 induces IL-8 expression through activation of the DNA damage response. PloS Pathog (2013) 9:e1003609. doi: 10.1371/journal.ppat.1003609 24068928 PMC3771893

[40] Benedict CA Butrovich KD Lurain NS Corbeil J Rooney I Schneider P. Cutting edge: a novel viral TNF receptor superfamily member in virulent strains of human cytomegalovirus. J Immunol (1999) 162:6967–70. doi: 10.4049/jimmunol.162.12.6967 10358135

[41] Cheung TC Humphreys IR Potter KG Norris PS Shumway HM Tran BR. Evolutionarily divergent herpesviruses modulate T cell activation by targeting the herpesvirus entry mediator cosignaling pathway. Proc Natl Acad Sci USA (2005) 102:13218–23. doi: 10.1073/pnas.0506172102 PMC1201609 16131544

[42] Bitra A Nemcovicova I Picarda G Doukov T Wang J Benedict CA. Structure of human cytomegalovirus UL144, an HVEM orthologue, bound to the B and T cell lymphocyte attenuator. J Biol Chem (2019) 294:10519–29. doi: 10.1074/jbc.RA119.009199 PMC6615696 31126984

[43] Poole E King CA Sinclair JH Alcami A. The UL144 gene product of human cytomegalovirus activates NFkappaB via a TRAF6-dependent mechanism. EMBO J (2006) 25:4390–9. doi: 10.1038/sj.emboj.7601287 PMC1570428 16932746

[44] Poole E Groves I MacDonald A Pang Y Alcami A Sinclair J. Identification of TRIM23 as a cofactor involved in the regulation of NF-kappaB by human cytomegalovirus. J Virol (2009) 83:3581–90. doi: 10.1128/JVI.02072-08 PMC2663253 19176615

[45] Weekes MP Tan SY Poole E Talbot S Antrobus R Smith DL. Latency-associated degradation of the MRP1 drug transporter during latent human cytomegalovirus infection. Science (2013) 340:199–202. doi: 10.1126/science.1235047 23580527 PMC3683642

[46] Montag C Wagner JA Gruska I Vetter B Wiebusch L Hagemeier C. The latency-associated UL138 gene product of human cytomegalovirus sensitizes cells to tumor necrosis factor alpha (TNF-alpha) signaling by upregulating TNF-alpha receptor 1 cell surface expression. J Virol (2011) 85:11409–21. doi: 10.1128/JVI.05028-11 PMC3194957 21880774

[47] Jakobsen PH Dodt KK Meyer CN Katzenstein T Gerstoft J Skinhoj P. Increased levels of soluble tumour necrosis factor receptor-I (P55) and decreased IgG1 reactivities in HIV-1 patients with cytomegalovirus disease. Scand J Immunol (1998) 47:591–5. doi: 10.1046/j.1365-3083.1998.00338.x 9652828

[48] Humbert M Roux-Lombard P Cerrina J Magnan A Simonneau G Dartevelle P. Soluble TNF receptors (TNF-sR55 and TNF-sR75) in lung allograft recipients displaying cytomegalovirus pneumonitis. Am J Respir Crit Care Med (1994) 149:1681–5. doi: 10.1164/ajrccm.149.6.8004330 8004330

[49] Le-Trilling VTK Becker T Nachshon A Stern-Ginossar N Scholer L Voigt S. The human cytomegalovirus pUL145 isoforms act as viral DDB1-cullin-associated factors to instruct host protein degradation to impede innate immunity. Cell Rep (2020) 30:2248–2260 e5. doi: 10.1016/j.celrep.2020.01.070 32075763

[50] Sinzger C Hahn G Digel M Katona R Sampaio KL Messerle M. Cloning and sequencing of a highly productive, endotheliotropic virus strain derived from human cytomegalovirus TB40/E. J Gen Virol (2008) 89:359–68. doi: 10.1099/vir.0.83286-0 18198366

[51] Hengel H Esslinger C Pool J Goulmy E Koszinowski UH. Cytokines restore MHC class I complex formation and control antigen presentation in human cytomegalovirus-infected cells. J Gen Virol (1995) 76(Pt 12):2987–97. doi: 10.1099/0022-1317-76-12-2987 8847504

[52] Wagner M Ruzsics Z Koszinowski UH. Herpesvirus genetics has come of age. Trends Microbiol (2002) 10:318–24. doi: 10.1016/S0966-842X(02)02394-6 12110210

[53] Tischer BK von Einem J Kaufer B Osterrieder N. Two-step red-mediated recombination for versatile high-efficiency markerless DNA manipulation in Escherichia coli. Biotechniques (2006) 40:191–7. doi: 10.2144/000112096 16526409

[54] Atalay R Zimmermann A Wagner M Borst E Benz C Messerle M. Identification and expression of human cytomegalovirus transcription units coding for two distinct Fcgamma receptor homologs. J Virol (2002) 76:8596–608. doi: 10.1128/JVI.76.17.8596-8608.2002 PMC136976 12163579

[55] Fischer R Maier O Naumer M Krippner-Heidenreich A Scheurich P Pfizenmaier K. Ligand-induced internalization of TNF receptor 2 mediated by a di-leucin motif is dispensable for activation of the NFkappaB pathway. Cell Signal (2011) 23:161–70. doi: 10.1016/j.cellsig.2010.08.016 20807567

[56] Schneider-Brachert W Tchikov V Neumeyer J Jakob M Winoto-Morbach S Held-Feindt J. Compartmentalization of TNF receptor 1 signaling: internalized TNF receptosomes as death signaling vesicles. Immunity (2004) 21:415–28. doi: 10.1016/j.immuni.2004.08.017 15357952

[57] Kanehisa M Furumichi M Sato Y Kawashima M Ishiguro-Watanabe M. KEGG for taxonomy-based analysis of pathways and genomes. Nucleic Acids Res (2023) 51:D587–92. doi: 10.1093/nar/gkac963 PMC9825424 36300620

[58] Tirosh O Cohen Y Shitrit A Shani O Le-Trilling VT Trilling M. The transcription and translation landscapes during human cytomegalovirus infection reveal novel host-pathogen interactions. PloS Pathog (2015) 11:e1005288. doi: 10.1371/journal.ppat.1005288 26599541 PMC4658056

[59] Aggarwal BB. Signalling pathways of the TNF superfamily: a double-edged sword. Nat Rev Immunol (2003) 3:745–56. doi: 10.1038/nri1184 12949498

[60] Nguyen CC Domma AJ Zhang H Kamil JP. Endoplasmic reticulum (ER) reorganization and intracellular retention of CD58 are functionally independent properties of the human cytomegalovirus ER-resident glycoprotein UL148. J Virol (2020) 94(5):e01435-19. doi: 10.1128/JVI.01435-19 31801856 PMC7022370

[61] Wang ECY Pjechova M Nightingale K Vlahava VM Patel M Ruckova E. Suppression of costimulation by human cytomegalovirus promotes evasion of cellular immune defenses. Proc Natl Acad Sci USA (2018) 115:4998–5003. doi: 10.1073/pnas.1720950115 29691324 PMC5948980

[62] Bell JH Herrera AH Li Y Walcheck B. Role of ADAM17 in the ectodomain shedding of TNF-alpha and its receptors by neutrophils and macrophages. J Leukoc Biol (2007) 82:173–6. doi: 10.1189/jlb.0307193 17510296

[63] Peschon JJ Slack JL Reddy P Stocking KL Sunnarborg SW Lee DC. An essential role for ectodomain shedding in mammalian development. Science (1998) 282:1281–4. doi: 10.1126/science.282.5392.1281 9812885

[64] Kuhnert P Kemper O Wallach D. Cloning, sequencing and partial functional characterization of the 5' region of the human p75 tumor necrosis factor receptor-encoding gene (TNF-R). Gene (1994) 150:381–6. doi: 10.1016/0378-1119(94)90457-X 7821811

[65] Santee SM Owen-Schaub LB. Human tumor necrosis factor receptor p75/80 (CD120b) gene structure and promoter characterization. J Biol Chem (1996) 271:21151–9. doi: 10.1074/jbc.271.35.21151 8702885

[66] Polz J Remke A Weber S Schmidt D Weber-Steffens D Pietryga-Krieger A. Myeloid suppressor cells require membrane TNFR2 expression for suppressive activity. Immun Inflamm Dis (2014) 2:121–30. doi: 10.1002/iid3.19 PMC4217546 25400932

[67] Beldi G Bahiraii S Lezin C Nouri Barkestani M Abdelgawad ME Uzan G. TNFR2 is a crucial hub controlling mesenchymal stem cell biological and functional properties. Front Cell Dev Biol (2020) 8:596831. doi: 10.3389/fcell.2020.596831 33344453 PMC7746825

[68] Zhang K Na T Wang L Gao Q Yin W Wang J. Human diploid MRC-5 cells exhibit several critical properties of human umbilical cord-derived mesenchymal stem cells. Vaccine (2014) 32:6820–7. doi: 10.1016/j.vaccine.2014.07.071 25086263

[69] Wang YP Qi Y Huang YJ Qi ML Ma YP He R. Identification of immediate early gene X-1 as a cellular target gene of hcmv-mir-UL148D. Int J Mol Med (2013) 31:959–66. doi: 10.3892/ijmm.2013.1271 23403649

[70] Lau B Poole E Krishna B Sellart I Wills MR Murphy E. The expression of human cytomegalovirus microRNA miR-UL148D during latent infection in primary myeloid cells inhibits activin A-triggered secretion of IL-6. Sci Rep (2016) 6:31205. doi: 10.1038/srep31205 27491954 PMC4974560

[71] Kim Y Lee S Kim S Kim D Ahn JH Ahn K. Human cytomegalovirus clinical strain-specific microRNA miR-UL148D targets the human chemokine RANTES during infection. PloS Pathog (2012) 8:e1002577. doi: 10.1371/journal.ppat.1002577 22412377 PMC3297591

[72] Babu SG Pandeya A Verma N Shukla N Kumar RV Saxena S. Role of HCMV miR-UL70-3p and miR-UL148D in overcoming the cellular apoptosis. Mol Cell Biochem (2014) 393:89–98. doi: 10.1007/s11010-014-2049-8 24737391

[73] Pandeya A Khalko RK Singh S Kumar M Gosipatala SB. Hcmv-miR-UL148D regulates the staurosporine-induced apoptosis by targeting the Endoplasmic Reticulum to Nucleus signaling 1(ERN1). PloS One (2022) 17:e0275072. doi: 10.1371/journal.pone.0275072 36156601 PMC9512192

[74] Pavelin J Reynolds N Chiweshe S Wu G Tiribassi R Grey F. Systematic microRNA analysis identifies ATP6V0C as an essential host factor for human cytomegalovirus replication. PloS Pathog (2013) 9:e1003820. doi: 10.1371/journal.ppat.1003820 24385903 PMC3873435

[75] Sijmons S Thys K Mbong Ngwese M Van Damme E Dvorak J Van Loock M. High-throughput analysis of human cytomegalovirus genome diversity highlights the widespread occurrence of gene-disrupting mutations and pervasive recombination. J Virol (2015) 89:7673–95. doi: 10.1128/JVI.00578-15 PMC4505652 25972543

[76] Li G Nguyen CC Ryckman BJ Britt WJ Kamil JP. A viral regulator of glycoprotein complexes contributes to human cytomegalovirus cell tropism. Proc Natl Acad Sci USA (2015) 112:4471–6. doi: 10.1073/pnas.1419875112 PMC4394275 25831500

[77] Siddiquey MNA Zhang H Nguyen CC Domma AJ Kamil JP. The human cytomegalovirus endoplasmic reticulum-resident glycoprotein UL148 activates the unfolded protein response. J Virol (2018) 92(20):e00896-18. doi: 10.1128/JVI.00896-18 30045994 PMC6158444

[78] Zhang H Read C Nguyen CC Siddiquey MNA Shang C Hall CM. The human cytomegalovirus nonstructural glycoprotein UL148 reorganizes the endoplasmic reticulum. mBio (2019) 10(6):e02110-19. doi: 10.1128/mBio.02110-19 31822584 PMC6904874

[79] Nguyen CC Siddiquey MNA Zhang H Li G Kamil JP. Human cytomegalovirus tropism modulator UL148 interacts with SEL1L, a cellular factor that governs endoplasmic reticulum-associated degradation of the viral envelope glycoprotein gO. J Virol (2018) 92(18):e00688-18. doi: 10.1128/JVI.00688-18 29997207 PMC6146704

[80] Wu CJ Conze DB Li X Ying SX Hanover JA Ashwell JD. TNF-alpha induced c-IAP1/TRAF2 complex translocation to a Ubc6-containing compartment and TRAF2 ubiquitination. EMBO J (2005) 24:1886–98. doi: 10.1038/sj.emboj.7600649 PMC1142588 15861135

[81] Oh RS Bai X Rommens JM. Human homologs of Ubc6p ubiquitin-conjugating enzyme and phosphorylation of HsUbc6e in response to endoplasmic reticulum stress. J Biol Chem (2006) 281:21480–90. doi: 10.1074/jbc.M601843200 16720581

[82] Li M Liu Y Xia F Wu Z Deng L Jiang R. Progranulin is required for proper ER stress response and inhibits ER stress-mediated apoptosis through TNFR2. Cell Signal (2014) 26:1539–48. doi: 10.1016/j.cellsig.2014.03.026 24703938

[83] Secchiero P Mirandola P Zella D Celeghini C Gonelli A Vitale M. Human herpesvirus 7 induces the functional up-regulation of tumor necrosis factor-related apoptosis-inducing ligand (TRAIL) coupled to TRAIL-R1 down-modulation in CD4(+) T cells. Blood (2001) 98:2474–81. doi: 10.1182/blood.V98.8.2474 11588045

[84] Popkin DL Virgin W.T. Murine cytomegalovirus infection inhibits tumor necrosis factor alpha responses in primary macrophages. J Virol (2003) 77:10125–30. doi: 10.1128/JVI.77.18.10125-10130.2003 PMC224571 12941924

[85] MacEwan DJ ligands TNF. and receptors–a matter of life and death. Br J Pharmacol (2002) 135:855–75. doi: 10.1038/sj.bjp.0704549 PMC1573213 11861313

[86] Suresh M Gao X Fischer C Miller NE Tewari K. Dissection of antiviral and immune regulatory functions of tumor necrosis factor receptors in a chronic lymphocytic choriomeningitis virus infection. J Virol (2004) 78:3906–18. doi: 10.1128/JVI.78.8.3906-3918.2004 PMC374248 15047807

[87] Wong GH Tartaglia LA Lee MS Goeddel DV. Antiviral activity of tumor necrosis factor is signaled through the 55-kDa type I TNF receptor [corrected]. J Immunol (1992) 149:3350–3. doi: 10.4049/jimmunol.149.10.3350 1331233

[88] Herbein G Gordon S. 55- and 75-kilodalton tumor necrosis factor receptors mediate distinct actions in regard to human immunodeficiency virus type 1 replication in primary human macrophages. J Virol (1997) 71:4150–6. doi: 10.1128/jvi.71.5.4150-4156.1997 PMC191574 9094699

[89] Herbein G Montaner LJ Gordon S. Tumor necrosis factor alpha inhibits entry of human immunodeficiency virus type 1 into primary human macrophages: a selective role for the 75-kilodalton receptor. J Virol (1996) 70:7388–97. doi: 10.1128/jvi.70.11.7388-7397.1996 PMC190806 8892857

[90] Roca FJ Mulero I Lopez-Munoz A Sepulcre MP Renshaw SA Meseguer J. Evolution of the inflammatory response in vertebrates: fish TNF-alpha is a powerful activator of endothelial cells but hardly activates phagocytes. J Immunol (2008) 181:5071–81. doi: 10.4049/jimmunol.181.7.5071 18802111

[91] Espin-Palazon R Martinez-Lopez A Roca FJ Lopez-Munoz A Tyrkalska SD Candel S. TNFalpha impairs rhabdoviral clearance by inhibiting the host autophagic antiviral response. PloS Pathog (2016) 12:e1005699. doi: 10.1371/journal.ppat.1005699 27351838 PMC4924823

[92] Hein MY Weissman JS. Functional single-cell genomics of human cytomegalovirus infection. Nat Biotechnol (2022) 40:391–401. doi: 10.1038/s41587-021-01059-3 34697476

[93] Lee CH Griffiths S Digard P Pham N Auer M Haas J. Asparagine deprivation causes a reversible inhibition of human cytomegalovirus acute virus replication. mBio (2019) 10(5):e01651-19. doi: 10.1128/mBio.01651-19 31594813 PMC6786868

[94] Chopra M Biehl M Steinfatt T Brandl A Kums J Amich J. Exogenous TNFR2 activation protects from acute GvHD via host T reg cell expansion. J Exp Med (2016) 213:1881–900. doi: 10.1084/jem.20151563 PMC4995078 27526711

[95] Torrey H Kuhtreiber WM Okubo Y Tran L Case K Zheng H. A novel TNFR2 agonist antibody expands highly potent regulatory T cells. Sci Signal (2020) 13(661):eaba9600. doi: 10.1126/scisignal.aba9600 33293464

[96] Sheng Y Li F Qin Z. TNF receptor 2 makes tumor necrosis factor a friend of tumors. Front Immunol (2018) 9:1170. doi: 10.3389/fimmu.2018.01170 29892300 PMC5985372

[97] Yang F Zhao N Wu N. TNFR2 promotes Adriamycin resistance in breast cancer cells by repairing DNA damage. Mol Med Rep (2017) 16:2962–8. doi: 10.3892/mmr.2017.6898 28677724

[98] Naserian S Abdelgawad ME Afshar Bakshloo M Ha G Arouche N Cohen JL. The TNF/TNFR2 signaling pathway is a key regulatory factor in endothelial progenitor cell immunosuppressive effect. Cell Commun Signal (2020) 18:94. doi: 10.1186/s12964-020-00564-3 32546175 PMC7298859

[99] Rubina A Patel M Nightingale K Potts M Fielding CA Kollnberger S. ADAM17 targeting by human cytomegalovirus remodels the cell surface proteome to simultaneously regulate multiple immune pathways. bioRxiv (2023) 2023:03. doi: 10.1101/2023.03.16.532955 PMC10438378 37561786

