# Supplementary material for: Deletion of the non-adjacent genes UL148 and UL148D impairs human cytomegalovirus-mediated TNF receptor 2 surface upregulation
Source: Front Immunol. 2023 Aug 3;14:1170300. doi: 10.3389/fimmu.2023.1170300 (PMC10437809; doi:10.3389/fimmu.2023.1170300)
Supplement: Supplementary file 1 [file Table_1.docx]

Supplementary Material

Deletion of the non-adjacent genes UL148 and UL148D impairs human cytomegalovirus-mediated TNF receptor 2 surface upregulation

Vu Thuy Khanh Le-Trilling, Fabienne Maaßen, Benjamin Katschinski, Hartmut Hengel, Mirko Trilling*

*** Correspondence:** Corresponding Author: Mirko.Trilling@uk-essen.de

# Supplementary Figures and Tables

**Supplementary Table S1:**

Oligonucleotides used for the construction of recombinant HCMV mutants

| **Oligonucleotide** | **Sequence (3´- 5´)** |
| --- | --- |
| AD169  ΔUL148-Kana1 | CTACCGACGCCGCGACACCAGGTAGGTTATCAAAACGCGAGCCCATATCGCCGCCATCATCCAGTGAATTCGAGCTCGGTAC |
| AD169  ΔUL148-Kana2 | GCTGGTAGCGTTTATGAGTCGGGCGGTGGCCGGCACGCCGCATTTCCTAACCCGCGCAGCGACCATGATTACGCCAAGCTCC |
| AD169  ΔUL147A-Kana1 | GGTTAGGAAATGCGGCGTGCCGGCCACCGCCCGACTCATAAACGCTACCAGCATGACGTTCCAGTGAATTCGAGCTCGGTAC |
| AD169  ΔUL147A-Kana2 | CTTTCTCTGGAAATACGCCAAGAAACTGAATTACCACTACTTTAGACTGCGCTGGTGATCGACCATGATTACGCCAAGCTCC |
| AD169  ΔUL147-Kana1 | TATACCACACCAGAGCGCTCAGTGTGCCCAGAGCTACCGCACGGTAAAATAGGGACATGACCAGTGAATTCGAGCTCGGTAC |
| AD169  ΔUL147-Kana2 | GTAATATGTGTATAGTTGATAAAATTTTTACAAGAAACGATAACGCAATATGTTTTCGATGACCATGATTACGCCAAGCTCC |
| AD169  ΔUL146-Kana1 | ACATATTGCGTTATCGTTTCTTGTAAAAATTTTATCAACTATACACATATTACTGATTCGCCAGTGAATTCGAGCTCGGTAC |
| AD169  ΔUL146-Kana2 | GTGATTTTCCGGGAATACCGGATATTACGAATTACTGATAGTGACGTAGCTAATAAAATTGACCATGATTACGCCAAGCTCC |
| AD169  ΔUL148A-Kana1 | TTTATCCCTACAGCGGCTGCCGAGTCACGTCCGCCGGCGCCCATCGGCCGCGGCGATCCCCAGTGAATTCGAGCTCGGTAC |
| AD169  ΔUL148A-Kana2 | GCCGCGCGACAACGGGCGAGCGGCGGTGAGACAGACGCGCGCACGTCGGCGACGATAGCCGACCATGATTACGCCAAGCTCC |
| AD169  ΔUL148B-Kana1 | GCTAGCCCGTCGCCGCCTCGGGGCACGGTGCCCTCCTACCCACGTAACTTCCTCCGTGACCCAGTGAATTCGAGCTCGGTAC |
| AD169  ΔUL148B-Kana2 | TGAAAACGGGGACTGTGGAGTTGTTGCTTTGTTCAGGAGACGACGACGGGAGCGAACGGGGACCATGATTACGCCAAGCTCC |
| AD169  ΔUL148C-Kana1 | CAAAGCAACAACTCCACAGTCCCCGTTTTCAACCGTTTTTGTTTCCTTCTCCGCGACTAGCCAGTGAATTCGAGCTCGGTAC |
| AD169  ΔUL148C-Kana2 | TGATTTCTTTTTTTCCTGTGTTAACACCGGCGTTTTCGGGACGGTCGGTTAACGTGGGTTGACCATGATTACGCCAAGCTCC |
| AD169  ΔUL148D-Kana1 | GTTTCCCTCGACTAGGGACCCGCCGAAGCAACTGCCGGAACAACCTGGAGGAGTCGCGGCCCAGTGAATTCGAGCTCGGTAC |
| AD169  ΔUL148D-Kana2 | CGTCGCGATCGGCAGGCGGGCCGGCTACGGCGCTTGGAGCTGTAGCCGCCACAAACTTGTGACCATGATTACGCCAAGCTCC |
| AD169  ΔUL139-Kana1 | TCACCGAGGCGGAGGTGGAAATGAGCCGTCCTGTGGGGGAGTGTACGACCCTGTAGTGCCCCAGTGAATTCGAGCTCGGTAC |
| AD169  ΔUL133-Kana2 | ATGGGTTGCGACGTGCACGATCCTTCGTGGCAATGCCAATGGGGCGTTCCCACGATTATT GACCATGATTACGCCAAGCTCC |
| AD169  ΔUL138-Kana1 | AGCAGAGAATGTCAAAGCGACATTATCGCGATCCGCTCCCCTCTTTTTTCTTTTTCTCATCCAGTGAATTCGAGCTCGGTAC |
| AD169  ΔUL138-Kana2 | GCGCGAGTGCTGTACAAAAGAGAGAGACTGGGACGTAGATCCGGACAGAGGACGGTCACCGACCATGATTACGCCAAGCTCC |
| AD169  ΔUL149-Kana1 | CACTACCTATGTGGTCAAGACCAAGGAACGGCCCTGGTGGCCCGACAACGCCATCAGGAGCCAGTGAATTCGAGCTCGGTAC |
| AD169  ΔUL150-Kana2 | CCGCTATTCTTTATTAACGTCTTATTCCCCCCGCTTCCACACACAACGCTGCATACAGCT GACCATGATTACGCCAAGCTCC |
| Δgpt-Kana1 | GCATGTTCCACATGTACGCGCTAGACGTGTAATCCACTCGCAGTTCGGGGACGCAACGCACCAGTGAATTCGAGCTCGGTAC |
| Δgpt-Kana2 | CGATGCAAGTGTGTCGCTGTCGAGTTTAAACATGCATCCTTAATTAAGGCTGCGATCTATCACCATGATTACGCCAAGCTCC |

**Supplementary Table S2:**

Oligonucleotides used for RT-PCR and generation of northernblot probes

| **Gene** | **Primer sequence (3´- 5´)** |
| --- | --- |
| TNFR1-1 | ACCAAGTGCCACAAAGGAAC |
| TNFR1-2 | CACACGGTGTTCTGTTTCTCC |
| TNFR2-1 | TGGCATTTACACCCTACGC |
| TNFR2-2 | GTTTCAGTTCCTGGTCTGGC |
| IkBa-1 | CATCCATGAAGAAAAGGCACT |
| IkBa-2 | TCAGGATTTTGCAGGTCCA |
| HCMV-IE1-1 | CTGGTCAGCCTTGCTTCTAGTCACC |
| HCMV-IE1-2 | TCTCCTAGTGTGGATGACCTACGGG |
| HCMV-IE2-1 | ACTTGTTCCTCAGGTCCTGGATGG |
| HCMV-IE2-2 | CCGATGCTTGTAACGAAGGCG |
| US9-1 | CGTCTTTAGCCTCTTCTTCCCGTG |
| US9-2 | GAAACTGAGCTCCCACAGGTGGA |
| UL139-1 | ATGCTGTGGATATTAGTTTTATTTGC |
| UL139-2 | CCGAGGCGGAGGTGGAAATG |
| UL111A-1 | TGCTGTCGGTGATGGTCTCTTCC |
| UL111A-2 | ACCGTACCTACGTGACCTACCAACG |
